# Knowledge, attitudes, and practices regarding venous thromboembolism prevention in patients with lung cancer

**DOI:** 10.3389/fpubh.2026.1980978

**Published:** 2026-10-13

**Authors:** Yuhang Ding, Yanhong Meng, Pengnan Xu, Jinping Zhang, Qiuhui Wu

**Affiliations:** 1 Department of Pharmacy, Nanjing Drum Tower Hospital, China Pharmaceutical University, Nanjing, China; 2 Department of Pharmacy, Nanjing Drum Tower Hospital, Affiliated Hospital of Medical School, Nanjing University, Nanjing, China

**Keywords:** cancer-associated thrombosis, health literacy, knowledge-attitude-practice, lung cancer, patient education, prevention, venous thromboembolism

## Abstract

**Background:**

Patients with lung cancer are at high risk of venous thromboembolism (VTE), yet their awareness and preventive behaviors remain insufficiently characterized. This study assessed knowledge, attitudes, and practices (KAP) regarding VTE prevention among patients with lung cancer and explored factors associated with these dimensions.

**Methods:**

This cross-sectional study included 244 patients with lung cancer recruited at Nanjing Drum Tower Hospital between January and May 2026. Participants completed a KAP questionnaire regarding VTE prevention. Univariable and multivariable logistic regression analyses were used to identify factors associated with KAP outcomes, and Pearson correlation analysis was used to examine the associations among knowledge, attitudes, and practices.

**Results:**

The mean knowledge, attitude, and practice scores were 8.98 ± 3.52, 31.18 ± 3.37, and 19.19 ± 4.49, respectively. Rural or suburban residence (adjusted OR = 0.447, 95% CI 0.201–0.992) and no previous VTE education (adjusted OR = 0.271, 95% CI 0.147–0.501) were associated with lower odds of meeting the operational knowledge threshold. Higher knowledge scores were associated with more favorable attitudes and higher self-reported preventive practice scores, while higher attitude scores were associated with higher self-reported preventive practice scores.

**Conclusion:**

Patients with lung cancer showed persistent gaps in VTE-related knowledge, generally favorable responses to several attitude items, and heterogeneous self-reported preventive behaviors. The findings highlight potential targets for patient education, particularly among rural or suburban residents and those without previous VTE education.

## Introduction

Venous thromboembolism (VTE), comprising deep vein thrombosis (DVT) and pulmonary embolism (PE), is a common and potentially life-threatening complication in patients with cancer (1). Among all malignancies, lung cancer is associated with a particularly high risk of VTE. A large retrospective study reported that the incidence of VTE was 13.9% among 6,732 patients with lung cancer, compared with only 1.4% among 17,284 individuals without lung cancer (2). Beyond its high incidence, VTE is also an important determinant of prognosis in this population. It is recognized as the second leading cause of death in patients with cancer after disease progression (2, 3), and its occurrence may increase the risk of mortality by two- to six-fold (4). Effective prevention of VTE in this population depends not only on appropriate thromboprophylaxis but also on patients' ability to recognize symptoms and adhere to preventive measures.

In addition to its clinical consequences, VTE imposes a substantial economic burden on healthcare systems worldwide. In the United States alone, the annual healthcare expenditure attributable to VTE has been estimated at $7–10 billion (5). Given its serious health and economic implications, prevention and early identification of VTE have become essential components of cancer care. Consequently, authoritative organizations, including the National Comprehensive Cancer Network (NCCN) (6) and the American Society of Clinical Oncology (ASCO) (7), recommend routine VTE risk assessment, patient education, and implementation of appropriate thromboprophylaxis strategies for patients with cancer. Despite these recommendations, however, awareness of cancer-associated thrombosis (CAT) among patients remains inadequate. A survey of 140 cancer patients in the United States found that 61% were unaware that cancer itself increases the risk of thrombosis (8). Moreover, patient education regarding VTE prevention is frequently overlooked in routine clinical practice, and data on lung cancer patients' specific knowledge gaps and behavioral patterns remain limited. From a public health education perspective, identifying gaps in VTE-related knowledge and preventive behavior is important for developing accessible and targeted health communication strategies for this high-risk population.

The Knowledge–Attitude–Practice (KAP) theory provides a useful framework for understanding health-related behavioral change, positing that knowledge acquisition influences attitudes and beliefs, which subsequently shape health behaviors (9). Although the KAP framework has been widely applied in studies of chronic disease management and health education (10–12), only a few studies have systematically examined the KAP of lung cancer patients regarding VTE prevention, leaving important questions about the relationships among these dimensions and their modifiable determinants unexplored. Therefore, this study aimed to develop a KAP-based questionnaire to assess the status of VTE-related knowledge, attitudes, and preventive practices among patients with lung cancer. In addition, we explored factors associated with each KAP dimension and examined the associations among knowledge, attitudes, and practices within the conceptual KAP framework. The findings may help identify potential targets for educational strategies aimed at improving VTE-related awareness and supporting appropriate preventive behaviors among patients with lung cancer.

## Materials and methods

### Study design and participants

This cross-sectional study was conducted at the Department of Respiratory and Critical Care Medicine of Nanjing Drum Tower Hospital between January 2026 and May 2026. Potentially eligible patients with lung cancer were identified through consecutive screening of the hospital electronic medical record system based on hospitalization or treatment records during the study period. Patients who met the eligibility criteria were subsequently contacted by telephone, informed about the study, and invited to participate. After verbal informed consent was obtained, the questionnaire link or QR code was sent to the participant via WeChat. The first page of the questionnaire reiterated the study purpose, procedures, voluntary nature of participation, and confidentiality of the collected information, and participants proceeded with the questionnaire only after confirming their willingness to participate. Inclusion criteria were: (1) age ≥18 years; (2) pathologically or clinically diagnosed lung cancer; (3) ability to communicate and complete the questionnaire independently or with technical assistance when necessary; and (4) informed consent and voluntary participation. Exclusion criteria were: (1) experiencing acute VTE at the time of the survey; (2) a history of psychiatric disorders or severe cognitive/communication impairments that precluded cooperation with the survey; and (3) refusal to participate or withdrawal from the study. The study was approved by the Ethics Committee of Nanjing Drum Tower Hospital (Approval No.: 2026-0587) and conducted in accordance with the Declaration of Helsinki. The requirement for written informed consent was waived by the Ethics Committee, which approved verbal informed consent obtained by telephone.

Based on the commonly used recommendation of 5–10 participants per questionnaire item (13), the recommended analyzable sample size for the 26-item questionnaire was estimated to be 130–260 participants. Allowing for an anticipated 10% rate of non-completion or invalid responses, approximately 145–289 participants would need to be recruited. Recruitment was conducted consecutively throughout the prespecified study period from January to May 2026. During this period, 259 patients were screened, and 244 completed the questionnaire and were included in the final analysis, which was within the recommended analyzable sample-size range.

### Questionnaire

The questionnaire was originally developed by the research team for the present study rather than adapted from or translated from a previously validated instrument. Item generation was primarily guided by the American Society of Clinical Oncology (ASCO) guideline on venous thromboembolism prophylaxis and treatment in patients with cancer (7) and the International Initiative on Thrombosis and Cancer (ITAC) clinical practice guidelines (14). The initial item pool was designed to cover clinically relevant aspects of VTE-related knowledge, attitudes, and preventive practices among patients with lung cancer. The draft questionnaire was subsequently reviewed by a multidisciplinary panel of six experts selected on the basis of their clinical or professional experience in lung cancer care, VTE prevention, medication management, and patient education. The panel comprised four specialists in respiratory and oncology medicine and two specialists in clinical pharmacy and nursing management. The experts reviewed the relevance, clinical appropriateness, clarity, comprehensibility, and coverage of the questionnaire items, and revisions were made according to their collective feedback.

A pilot study involving 30 patients with lung cancer was conducted before the formal survey to assess feasibility, item clarity, comprehensibility, and preliminary internal consistency. The overall Cronbach's α coefficient was 0.832. Domain-specific reliability analyses showed a Kuder–Richardson 20 (KR-20) coefficient of 0.788 for the knowledge domain, which was scored dichotomously as correct versus incorrect/uncertain, and Cronbach's α coefficients of 0.654 and 0.800 for the attitude and practice domains, respectively.

The final Chinese-language questionnaire consisted of four sections: demographic and clinical characteristics, knowledge, attitude, and practice. The knowledge dimension included 13 questions scored as 1 point for correct answers and 0 points for incorrect or uncertain responses, with a possible score range of 0–13 points. The attitude dimension included 8 questions using a five-point Likert scale. Positive attitude items (Questions 1–3 and 6–8) were scored from “strongly agree” (5 points) to “strongly disagree” (1 point), whereas two negatively worded items (Questions 4 and 5) were reverse-scored, with a possible score range of 8–40 points. The practice dimension included 5 questions and utilized a five-point Likert scale ranging from “always” (5 points) to “never” (1 point), with a possible score range of 5–25 points. Consistent with previous KAP studies (12, 15–17), a threshold of ≥70% of the maximum possible score was prespecified as a literature-based operational criterion. The corresponding integer cutoffs were ≥10 of 13 points for knowledge, ≥28 of 40 points for attitude, and ≥18 of 25 points for practice. Items concerning early ambulation, graduated compression stockings, anticoagulant prophylaxis, and bleeding monitoring were conditionally worded using phrases such as “when permitted by a physician,” “as prescribed,” or “during anticoagulant therapy.” Accordingly, these items were intended to assess self-reported engagement in clinician-directed preventive measures rather than the universal use, clinical indication, or guideline concordance of thromboprophylaxis. An English translation of the complete questionnaire used in this study is provided in Supplementary File S2.

The self-designed questionnaire was uploaded to the Wenjuanxing online survey platform (https://www.wjx.cn/), and the questionnaire link or QR code was distributed to potential participants via WeChat. Potentially eligible patients were contacted by telephone before questionnaire distribution. During the telephone contact, investigators explained the purpose and procedures of the study, the voluntary nature of participation, and the confidentiality of the collected information, and verbal informed consent was obtained. Patients who declined to participate were not provided access to the questionnaire. Those who agreed to participate received the questionnaire link or QR code via WeChat and completed the survey using a smartphone or tablet. When necessary, a family member could provide technical assistance with operating the device or entering responses according to the participant's instructions; however, all responses were required to reflect the participant's own answers, and family members were not permitted to answer questionnaire items on the participant's behalf. All questionnaire items were mandatory, and participants were required to complete all items before submission. Participants who accessed the questionnaire but did not complete and submit it were considered non-completers. To prevent duplicate submissions, each electronic device was restricted to one response. All submitted questionnaires were reviewed by the research team for data quality and completeness.

### Statistical analysis

IBM SPSS Statistics version 26.0 (IBM Corp., Armonk, NY, USA) was used for statistical analyses. Continuous variables, including knowledge, attitude, and practice scores, were expressed as mean ± standard deviation (SD). Comparisons between two groups were performed using independent-samples t tests, whereas comparisons among multiple groups were performed using one-way analysis of variance (ANOVA). Categorical variables were presented as frequencies (n) and percentages (%).

Pearson correlation analysis was used to assess the correlations among the continuous knowledge, attitude, and practice scores. To preserve the information contained in the original scores, continuous KAP scores were retained for descriptive, group-comparison, and correlation analyses. The ≥70% threshold was applied only for secondary exploratory logistic regression analyses, in which meeting the operational thresholds for adequate knowledge, positive attitude, and self-reported preventive practice was used as the binary dependent outcome. Univariable logistic regression analyses were first performed. Multivariable models were constructed by considering the univariable findings together with clinical relevance, potential confounding, and the conceptual KAP framework, rather than relying solely on a univariable P-value threshold. The knowledge model included residence, occupation, educational level, medical insurance status, comorbidity burden, previous VTE education, and primary caregiver. The attitude model included knowledge score, comorbidity burden, reason for hospitalization, and previous VTE education. The practice model included knowledge score, attitude score, occupation, comorbidity burden, and previous VTE education. Multicollinearity was assessed using variance inflation factor (VIF) values. The multivariable logistic regression analyses were considered secondary exploratory analyses. Among the 244 participants, 130 vs. 114, 194 vs. 50, and 172 vs. 72 participants met versus did not meet the operational thresholds for adequate knowledge, positive attitude, and self-reported preventive practice, respectively. Model complexity was restricted with consideration of the number of participants in the smaller outcome category and the number of estimated model parameters. Because several categorical subgroups contained relatively few participants, estimates involving these categories were interpreted cautiously. All questionnaire items were mandatory, and only completed questionnaires were included in the analysis; therefore, there were no item-level missing data. A two-sided *P* < 0.05 was considered statistically significant.

## Results

During the study period, 259 patients with lung cancer were screened for eligibility. Of these, 9 declined to participate and 6 accessed the questionnaire but did not complete it. Consequently, 244 participants completed the questionnaire and were included in the final analysis, corresponding to an overall response rate of 94.21% (244/259). Among them, 140 (57.4%) were male, 175 (71.7%) lived in urban areas, 102 (41.8%) had an associate degree or above, and 227 (93.0%) were covered by medical insurance. Most participants were married (90.2%), and 124 (50.8%) reported no comorbidities. In addition, 101 patients (41.4%) had previously received education regarding venous thromboembolism (VTE) prevention.

The mean knowledge, attitude, and practice scores were 8.98 ± 3.52 (range: 0–13), 31.18 ± 3.37 (range: 8–40), and 19.19 ± 4.49 (range: 5–25), respectively. Knowledge scores differed significantly according to gender (*P* = 0.037), age (*P* = 0.035), residence (*P* < 0.001), educational level (*P* < 0.001), occupation (*P* = 0.011), medical insurance status (*P* = 0.020), number of comorbidities (*P* = 0.005), and previous VTE education (*P* < 0.001). Attitude scores were significantly associated with comorbidity burden (*P* = 0.045) and reason for hospitalization (*P* = 0.013). Practice scores varied significantly according to gender (*P* = 0.049), occupation (*P* = 0.042), comorbidity burden (*P* = 0.009), previous VTE education (*P* = 0.002), and primary caregiver (*P* = 0.042) (Table 1). These subgroup comparisons were exploratory and should be interpreted cautiously.

**Table 1:** Demographic characteristics and KAP scores.

Variables	*N* (%)	Knowledge mean ±SD	*P*	Attitude, mean ±SD	*P*	Practice, mean ±SD	*P*
***N*** **=** **244**	244						
**Total score**		8.98 ± 3.52		31.18 ± 3.37		19.19 ± 4.49	
**Gender**			**0.037**		0.813		**0.049**
Male	140 (57.4)	8.57 ± 3.73		31.23 ± 3.53		18.72 ± 4.86	
Female	104 (42.6)	9.52 ± 3.15		31.13 ± 3.16		19.82 ± 3.87	
**Age, years**			**0.035**		0.356		0.103
< 40	38 (15.6)	9.87 ± 2.92		31.08 ± 3.11		19.32 ± 3.98	
40~59	89 (36.5)	9.45 ± 3.71		31.61 ± 3.71		19.96 ± 4.77	
60~74	96 (39.3)	8.22 ± 3.48		31.04 ± 3.33		18.34 ± 4.60	
≥75	21 (8.6)	8.81 ± 3.40		30.24 ± 2.36		19.57 ± 3.09	
**Residence**			**< 0.001**		0.652		0.065
Urban	175 (71.7)	9.49 ± 3.27		31.25 ± 3.21		19.56 ± 4.12	
Rural or suburban	69 (28.3)	7.67 ± 3.81		31.03 ± 3.78		18.25 ± 5.23	
**Marital status**			0.784		0.544		0.295
Unmarried	18 (7.4)	9.11 ± 3.10		31.00 ± 2.68		19.33 ± 3.96	
Married	220 (90.2)	8.99 ± 3.53		31.16 ± 3.43		19.10 ± 4.56	
Others	6 (2.5)	8.00 ± 4.78		32.67 ± 3.27		22.00 ± 2.53	
**Education level**			**< 0.001**		0.783		0.114
Primary or below	39 (16.0)	8.03 ± 3.68		31.56 ± 3.77		18.38 ± 4.53	
Junior high school	53 (21.7)	7.51 ± 3.79		30.7 ± 3.52		18.09 ± 5.02	
Senior high school	50 (20.5)	9.20 ± 3.25		31.34 ± 2.74		20.06 ± 3.33	
Associate degree	40 (16.4)	9.80 ± 3.47		31.13 ± 4.01		19.43 ± 5.06	
Bachelor's or above	62 (25.4)	10.11 ± 2.89		31.27 ± 3.06		19.77 ± 4.30	
**Occupation**			**0.011**		0.128		**0.042**
Farmer	70 (28.7)	7.97 ± 3.90		31.43 ± 3.79		18.01 ± 4.91	
Worker	47 (19.3)	8.98 ± 3.40		30.83 ± 3.03		19.60 ± 3.55	
Civil servant	44 (18.0)	10.39 ± 3.47		31.57 ± 3.72		20.43 ± 5.34	
Self-employed	28 (11.5)	9.14 ± 2.89		32.18 ± 2.55		20.07 ± 3.72	
Retired	55 (22.5)	9.04 ± 3.14		30.36 ± 3.05		18.89 ± 4.00	
**Medical insurance**			**0.020**		0.742		0.063
Yes	227 (93.0)	9.12 ± 3.49		31.16 ± 3.28		19.33 ± 4.53	
No	17 (7.0)	7.06 ± 3.45		31.53 ± 4.48		17.24 ± 3.41	
**Other comorbidities**			**0.005**		**0.045**		**0.009**
0	124 (50.8)	9.32 ± 3.36		31.21 ± 3.43		19.53 ± 4.61	
1~2	100 (41.0)	9.02 ± 3.44		31.50 ± 3.31		19.35 ± 4.09	
≥ 3	20 (8.2)	6.60 ± 4.09		29.45 ± 2.98		16.25 ± 4.88	
**History of thrombosis**			0.723		0.728		0.203
Yes	35 (14.3)	9.17 ± 3.17		31.00 ± 3.22		20.09 ± 3.90	
No	209 (85.7)	8.94 ± 3.58		31.22 ± 3.40		19.04 ± 4.57	
**Activity status (past 3 days)**			0.328		0.972		0.610
Independent daily living	223 (91.4)	8.99 ± 3.52		31.17 ± 3.33		19.11 ± 4.50	
Require assistance	17 (7.0)	9.41 ± 3.57		31.29 ± 3.93		19.76 ± 4.21	
Completely bedridden	4 (1.6)	6.50 ± 3.42		31.50 ± 4.51		21.00 ± 6.16	
**Ever learned VTE**			**< 0.001**		0.199		**0.002**
Yes	101 (41.4)	10.14 ± 3.09		31.51 ± 3.20		20.27 ± 3.79	
No	143 (58.6)	8.15 ± 3.58		30.95 ± 3.48		18.43 ± 4.79	
**Primary caregiver**			0.105		0.385		**0.042**
Children	68 (27.9)	8.53 ± 3.33		31.09 ± 3.34		19.53 ± 4.03	
Spouse	80 (32.8)	8.53 ± 3.58		31.16 ± 3.25		18.30 ± 4.68	
Parents	44 (18.0)	10.14 ± 3.46		31.89 ± 3.64		19.89 ± 4.84	
Rotating care	13 (5.3)	9.54 ± 3.28		31.69 ± 2.69		21.92 ± 3.43	
Other	39 (16.0)	9.18 ± 3.71		30.44 ± 3.58		18.72 ± 4.42	
**Reason for hospitalization**			0.509		**0.013**		0.082
Lung cancer surgery	55 (22.5)	8.53 ± 3.53		30.91 ± 3.50		19.22 ± 4.65	
Systemic cancer therapy	99 (40.6)	9.06 ± 3.60		31.75 ± 3.14		18.77 ± 4.50	
Complication/symptom management	20 (8.2)	9.90 ± 2.75		32.35 ± 2.78		21.60 ± 3.38	
Routine follow-up	70 (28.7)	8.94 ± 3.62		30.27 ± 3.56		19.07 ± 4.52	

Bold values indicate statistically significant results (*P* < 0.05).

The distribution of knowledge items revealed that the three questions with the highest correct response rates were: “Which conditions should prompt notification of healthcare providers to rule out pulmonary embolism?” (K11, 87.7%), “Prolonged sitting or bed rest increases the risk of VTE” (K6, 82.4%), and “VTE can be prevented” (K8, 79.9%). In contrast, the lowest correct response rates were observed for “Recommended timing for wearing medical compression stockings” (K12, 45.1%), “VTE mainly includes deep vein thrombosis and pulmonary embolism” (K1, 54.5%), and “Cancer increases the risk of VTE” (K5, 63.5%) (Table 2).

**Table 2:** Distribution of correctness in knowledge.

Knowledge	Correctness rate, *n* (%)
1. Venous thromboembolism (VTE) mainly includes deep vein thrombosis and pulmonary embolism	133 (54.5)
2. Hypercoagulable states (blood that clots more easily than usual) is one of the main causes of VTE	164 (67.2)
3. VTE can present as unilateral lower extremity swelling and pain	154 (63.1)
4. Pulmonary embolism can present as dyspnea, chest pain, and hemoptysis, and can be life-threatening when severe	178 (73.0)
5. Cancer increases the risk of VTE	155 (63.5)
6. Prolonged sitting or bed rest increases the risk of VTE	201 (82.4)
7. Dislodgement of a deep vein thrombus may cause pulmonary embolism, which can be fatal	178 (73.0)
8. Do you think VTE can be prevented?	195 (79.9)
9. Wearing compression stockings, using pneumatic compression devices, or using anticoagulants can prevent VTE	156 (63.9)
10. VTE prevention/treatment (especially with anticoagulants) may increase the risk of bleeding, requiring monitoring and timely reporting of abnormal bleeding	167 (68.4)
11. Which of the following conditions should prompt you to notify healthcare providers to rule out pulmonary embolism?	214 (87.7)
12. When is the recommended time to wear medical compression stockings?	110 (45.1)
13. Which of the following bleeding signs is more indicative of “need for prompt reporting”?	185 (75.8)

Regarding attitudes toward VTE prevention, most participants agreed or strongly agreed that VTE prevention would provide health benefits (88.9%, A6), that they would actively cooperate with treatment and follow-up if diagnosed with VTE (94.7%, A7), and that they were confident in adhering to VTE prevention measures (91.4%, A8). However, concerns regarding anticoagulant-related bleeding remained common, with 40.6% of participants agreeing or strongly agreeing that fear of bleeding might reduce their willingness to use anticoagulants (A4) (Table 3).

**Table 3:** Distribution of attitudes.

Attitude	Strongly agree	Agree	Neutral	Disagree	Strongly disagree
	*n* (%)	*n* (%)	*n* (%)	*n* (%)	*n* (%)
1. I worry that I might develop VTE	71 (29.1)	70 (28.7)	77 (31.6)	19 (7.8)	7 (2.9)
2. I think that VTE will affect my physical health	114 (46.7)	98 (40.2)	24 (9.8)	7 (2.9)	1 (0.4)
3. I think that developing VTE will impose a financial burden on my family	119 (48.8)	105 (43.0)	14 (5.7)	5 (2.0)	1 (0.4)
4. I worry that using anticoagulants may cause bleeding, so I might be reluctant to use them	51 (20.9)	48 (19.7)	90 (36.9)	47 (19.3)	8 (3.3)
5. I find compression stockings or pneumatic compression devices uncomfortable/troublesome, so I find it difficult to adhere to their use	50 (20.5)	53 (21.7)	81 (33.2)	50 (20.5)	10 (4.1)
6. I believe that VTE prevention will bring health benefits to me	131 (53.7)	86 (35.2)	21 (8.6)	5 (2.0)	1 (0.4)
7. If I develop VTE, I will actively cooperate with healthcare providers for treatment and follow-up	158 (64.8)	73 (29.9)	11 (4.5)	2 (0.8)	0 (0.0)
8. I am confident that I can comply with the measures for VTE prevention	141 (57.8)	82 (33.6)	20 (8.2)	1 (0.4)	0 (0.0)

Participants demonstrated varying levels of self-reported VTE-related preventive behaviors. Early ambulation when permitted by physicians was reported as “always” or “often” by 82.8% of participants (P2). For treatment-related items, 81.1% reported monitoring for bleeding during anticoagulant therapy and notifying healthcare professionals of abnormalities (P5), while 73.8% reported adherence to prescribed anticoagulant therapy (P4). In contrast, only 35.7% routinely sought VTE-related information from healthcare professionals (P1) (Table 4).

**Table 4:** Distribution of practice.

Practice	Always	Often	Sometimes	Rarely	Never
	*n* (%)	*n* (%)	*n* (%)	*n* (%)	*n* (%)
1. Do you actively ask doctors, nurses, or pharmacists about knowledge of VTE prevention?	50 (20.5)	37 (15.2)	64 (26.2)	45 (18.4)	48 (19.7)
2. When permitted by the doctor, do you get out of bed and ambulate or increase walking as early as possible?	97 (39.8)	105 (43.0)	28 (11.5)	7 (2.9)	7 (2.9)
3. Do you comply with the use of medical graduated compression stockings for VTE prevention as prescribed?	88 (36.1)	76 (31.1)	45 (18.4)	13 (5.3)	22 (9.0)
4. Can you adhere to oral or injectable anticoagulant therapy for VTE prevention as prescribed?	114 (46.7)	66 (27.0)	35 (14.3)	16 (6.6)	13 (5.3)
5. During anticoagulant therapy, do you proactively monitor adverse reactions (e.g., gingival bleeding, conjunctival congestion, melena, etc.) and promptly inform the doctor, nurse, or pharmacist if any occur?	137 (56.1)	61 (25.0)	26 (10.7)	6 (2.5)	14 (5.7)

Correlation analysis demonstrated significant positive associations among all three KAP dimensions. Knowledge was positively correlated with attitude (*r* = 0.298, *P* < 0.001) and practice (*r* = 0.488, P < 0.001). Likewise, attitude was positively associated with practice (*r* = 0.442, *P* < 0.001) (Table 5).

**Table 5:** Correlation analysis between knowledge, attitude, and practice.

Variables	Knowledge	Attitude	Practice
Knowledge	1		
Attitude	0.298 (** < 0.001**)	1	
Practice	0.488 (** < 0.001**)	0.442 (** < 0.001**)	1

Bold values indicate statistically significant results (*P* < 0.05).

Using the prespecified literature-based operational threshold of ≥70%, 130 (53.3%), 194 (79.5%), and 172 (70.5%) participants met the operational thresholds for adequate knowledge, positive attitude, and self-reported preventive practice, respectively. Given the volume of data, the detailed results of the univariable logistic regression analyses for the three KAP outcomes are provided in Supplementary Table S1. Multivariable models were constructed based on the univariable findings together with clinical relevance, potential confounding, and the conceptual KAP framework, as described in the Statistical Analysis section.

Multivariable logistic regression analysis identified several factors independently associated with KAP outcomes (Table 6). In the multivariable knowledge model, participants living in rural or suburban areas were less likely to have adequate VTE-related knowledge than urban residents (OR = 0.447, 95% CI 0.201–0.992, *P* = 0.046). Those who had not previously received VTE education were also less likely to have adequate knowledge (OR = 0.271, 95% CI 0.147–0.501, *P* < 0.001).

**Table 6:** Multivariable analysis for knowledge, attitude, and practice.

Variables	Knowledge		Attitude		Practice	
	OR (95% CI)	*P*	OR (95% CI)	*P*	OR (95% CI)	*P*
**Knowledge**			**1.158 (1.049–1.279)**	**0.004**	**1.168 (1.062–1.285)**	**0.001**
**Attitude**					**1.233 (1.110–1.370)**	**< 0.001**
Residence						
Urban	ref		–	–	–	–
Rural or suburban	**0.447 (0.201–0.992)**	**0.046**	–	–	–	–
Education level						
Primary or below	ref					
Junior high school	0.547 (0.203–1.472)	0.233	–	–		
Senior high school	0.946 (0.322–2.775)	0.919	–	–		
Associate degree	2.311 (0.663–8.052)	0.188	–	–		
Bachelor's or above	0.826 (0.215–3.175)	0.781	–	–		
Occupation						
Farmer	ref		–	–	ref	
Worker	0.389 (0.128–1.179)	0.095	–	–	2.432 (0.954**–**6.199)	0.063
Civil servant	0.969 (0.259–3.622)	0.963	–	–	2.088 (0.738**–**5.906)	0.165
Self–employed	0.407 (0.121–1.377)	0.148	–	–	1.808 (0.589**–**5.547)	0.301
Retired	0.582 (0.205–1.651)	0.309	–	–	1.665 (0.704**–**3.940)	0.246
Medical insurance						
Yes	ref		–	–	–	–
No	0.303 (0.090–1.024)	0.055	–	–	–	–
Other comorbidities						
0	ref		ref		ref	
1~2	1.496 (0.782–2.863)	0.224	1.358 (0.646–2.856)	0.420	1.020 (0.513**–**2.025)	0.956
≥3	0.420 (0.125–1.408)	0.160	**0.282 (0.092–0.862)**	**0.026**	0.575 (0.186**–**1.779)	0.337
Ever learned VTE						
Yes	ref		ref		ref	
No	**0.271 (0.147–0.501)**	**< 0.001**	1.061 (0.505–2.231)	0.876	0.848 (0.430**–**1.671)	0.633
Primary caregiver						
Children	ref		–	–	–	–
Spouse	0.597 (0.264**–**1.348)	0.215	–	–	–	–
Parents	1.569 (0.549**–**4.478)	0.400	–	–	–	–
Rotating care	2.550 (0.601**–**10.825)	0.204	–	–	–	–
Other	1.342 (0.503**–**3.579)	0.557	–	–	–	–
Reason for hospitalization						
Lung cancer surgery	–		ref		–	–
Systemic cancer therapy	–		**2.888 (1.173–7.114)**	**0.021**	–	–
Complication/symptom management	–		5.653 (0.674**–**47.420)	0.110	–	–
Routine follow–up	–		0.791 (0.342**–**1.832)	0.585	–	–

Bold values indicate statistically significant results (*P* < 0.05).

For attitude, higher knowledge scores were associated with a higher likelihood of positive attitudes toward VTE prevention (OR = 1.158, 95% CI 1.049–1.279, *P* = 0.004). Having three or more comorbidities was associated with a lower likelihood of positive attitudes (OR = 0.282, 95% CI 0.092–0.862, *P* = 0.026). Hospitalization for systemic cancer therapy was associated with a higher likelihood of positive attitudes compared with hospitalization for lung cancer surgery (OR = 2.888, 95% CI 1.173–7.114, *P* = 0.021).

Regarding preventive practice, both higher knowledge scores (OR = 1.168, 95% CI 1.062–1.285, *P* = 0.001) and higher attitude scores (OR = 1.233, 95% CI 1.110–1.370, *P* < 0.001) were independently associated with meeting the operational threshold for self-reported preventive practice. No evidence of problematic multicollinearity was observed, with VIF values ranging from 1.044 to 1.620 across the multivariable models.

## Discussion

To our knowledge, this may be among the first studies to investigate knowledge, attitudes, and practices (KAP) regarding venous thromboembolism (VTE) prevention among patients with lung cancer using a KAP theoretical framework. Overall, patients with lung cancer demonstrated moderate levels of knowledge, relatively positive attitudes, and varying levels of self-reported preventive practices regarding VTE prevention. Significant positive associations were observed among the three KAP dimensions, with higher knowledge scores associated with more favorable attitudes and higher self-reported preventive practice scores, and more favorable attitudes associated with higher self-reported preventive practice scores. These findings were broadly consistent with the conceptual KAP framework, although the cross-sectional design precludes conclusions regarding temporal ordering or causality.

The overall knowledge score indicated substantial gaps in VTE prevention. Although most participants correctly recognized common risk factors (e.g., prolonged immobility) and symptoms of pulmonary embolism, important misconceptions persisted. Only 54.5% knew that VTE includes both deep vein thrombosis and pulmonary embolism, and fewer than two-thirds were aware that cancer itself is a major risk factor. These findings align with previous surveys: Bueno et al. reported that over half of cancer patients were unaware of cancer-related thrombotic risk, a Canadian survey found only 26.9% had received CAT education and 38.1% had no CAT knowledge, and a global survey (42 countries, *n* = 2,262) showed 55.3% received no VTE education during their cancer journey, with an additional 8.2% educated only at diagnosis (8, 18, 19). Potential explanations proposed in previous literature include limited opportunities for thrombosis education during routine oncology care, psychological burden, and patients' prioritization of cancer-related concerns over thrombotic risk. However, these factors were not directly assessed in the present study, and their contribution to the observed knowledge gaps remains uncertain.

First, rural and suburban residents exhibited lower VTE knowledge than urban patients. Previous studies have reported associations of rural residence with lower health literacy and reduced access to health information (20, 21); however, these factors were not directly assessed in the present study and therefore cannot be considered explanations for the observed association. The present finding should also be interpreted cautiously given the borderline statistical significance of the estimate. Second, previous VTE education remained strongly associated with adequate VTE-related knowledge. Only 41.4% of participants had received prior VTE education. Although previous intervention research has reported improvements in VTE-related awareness following educational interventions (22), the cross-sectional association observed in the present study does not establish that previous VTE education resulted in higher knowledge levels.

Most participants demonstrated positive attitudes toward VTE prevention. The majority agreed that VTE prevention provides health benefits (88.9%), and 94.7% expressed willingness to actively cooperate with treatment and follow-up if diagnosed with VTE. However, concerns regarding anticoagulant-related bleeding remained prevalent; nearly two-fifths of participants reported that fear of bleeding would lower their willingness to undergo anticoagulant treatment. Similar concerns have been observed in studies of cancer-associated thrombosis, where fear of treatment-related adverse events frequently influenced treatment acceptance and adherence (8, 14, 23). When anticoagulation is clinically indicated, healthcare professionals should provide balanced education regarding its potential benefits and bleeding risks to support informed decision-making and adherence to prescribed therapy.

An inverse association was observed between having three or more comorbidities and positive attitudes toward VTE prevention; however, this finding should be interpreted cautiously because the subgroup was small. Hospitalization for systemic anticancer therapy was also associated with more positive attitudes than hospitalization for surgery. The mechanisms underlying these associations were not assessed in the present study, and the relatively small subgroup sizes and imprecision of some estimates preclude firm conclusions. Differences in disease burden, treatment experience, and interactions with healthcare professionals may warrant evaluation in future studies.

Regarding self-reported preventive practices, relatively frequent engagement was observed for several clinician-directed behaviors, including early ambulation when permitted, adherence to prescribed anticoagulant therapy, and monitoring for bleeding during anticoagulant therapy. Importantly, these findings should not be interpreted as indicating that anticoagulant prophylaxis, graduated compression stockings, or other prophylactic measures are universally appropriate for all patients with lung cancer, because their use depends on individualized assessment of thrombotic and bleeding risks. Accordingly, the practice domain primarily reflects patients' self-reported adherence to or engagement in context-specific preventive behaviors rather than the clinical appropriateness or guideline concordance of thromboprophylaxis. However, only 35.7% of participants routinely sought VTE-related information from healthcare professionals, suggesting limited engagement in proactive health information acquisition. Previous studies have shown that active information-seeking behavior is associated with better self-management and adherence to preventive recommendations among patients with chronic diseases (24–26). The limited information-seeking observed in this study co-occurred with a moderate level of VTE knowledge; however, the cross-sectional design does not permit determination of the direction or mechanism of this relationship.

Multivariable analysis showed that both knowledge and attitude scores were independently associated with preventive practice. The correlation and multivariable logistic regression results were broadly consistent with the hypothesized association pattern of the KAP framework, with higher knowledge scores associated with more favorable attitudes and higher self-reported preventive practice scores, and more favorable attitudes associated with higher self-reported preventive practice scores. However, because all KAP dimensions were assessed simultaneously, these findings do not establish temporal ordering or causal relationships. The moderate correlations among the KAP dimensions also suggest that preventive practices may be associated with additional factors, including motivation, self-efficacy, social support, and access to healthcare resources (27, 28).

From a public health education perspective, these findings highlight the need for VTE communication strategies that are targeted and accessible across different residential and sociodemographic groups. Future VTE prevention interventions should adopt multi-dimensional strategies. The present study provides novel insights by focusing specifically on patients with lung cancer—one of the malignancies at highest risk for CAT—and by combining KAP assessment with correlation and multivariable analyses to characterize the relationships among knowledge, attitudes, and preventive practices. Future interventions could evaluate structured health education, shared decision-making, and targeted support for practical barriers as potential strategies to improve VTE-related knowledge, attitudes, and preventive practices. Notably, an IMB model-based intervention study in older patients with lung cancer reported that theory-guided health education was associated with improved VTE preventive behaviors and a lower incidence of VTE, suggesting the potential clinical value of such standardized educational programs (22).

Several limitations should be acknowledged. First, the cross-sectional design precludes causal inference regarding the relationships among knowledge, attitudes, and practices because all variables were measured at the same time point. Reverse causation cannot be excluded, and residual or unmeasured confounding may also partly account for the observed associations. Second, the study was conducted at a single tertiary hospital, which may limit the representativeness and generalizability of the findings to patients in other regions or healthcare settings. Several categorical subgroups were also small, which may have increased the possibility of chance findings and reduced the precision and stability of some regression estimates, particularly where confidence intervals were wide; these associations should therefore be interpreted cautiously. Third, although potentially eligible patients were identified through electronic medical records, participation required subsequent telephone contact and electronic questionnaire completion via WeChat and the online survey platform. This recruitment and survey process may have underrepresented patients with limited digital access or literacy, particularly older, rural, or less-educated individuals, and may therefore have influenced the observed KAP levels and associations. Fourth, the use of self-reported questionnaires may have introduced recall bias and social desirability bias. Fifth, the psychometric validation of the study-specific questionnaire remains preliminary, as formal content and construct validity analyses were not performed and the attitude domain showed comparatively modest internal consistency (Cronbach's α = 0.654). Further validation in larger independent populations is warranted. Sixth, the study did not verify the clinical indication or guideline concordance of individual prophylactic measures, objectively assess adherence, or evaluate subsequent VTE, bleeding, or other clinical outcomes. Therefore, self-reported practice scores should not be interpreted as evidence of appropriate thromboprophylaxis or reduced VTE risk.

## Conclusion

Patients with lung cancer showed persistent gaps in VTE-related knowledge, generally favorable responses to several attitude items, and heterogeneous self-reported preventive behaviors. Significant associations were observed among knowledge, attitudes, and practices, broadly consistent with the conceptual KAP framework in this population. These findings may help identify patient groups and educational domains that warrant greater attention, particularly among rural or suburban residents and those without prior VTE education. Whether improvements in KAP translate into more guideline-concordant thromboprophylaxis or better clinical outcomes requires prospective investigation.

## Data Availability

The datasets generated and/or analyzed during the current study are not publicly available due to concerns regarding participant confidentiality and potentially identifiable clinical information. Requests to access the datasets should be directed to the corresponding author and will be considered subject to institutional and ethical approval.
