# Supplementary material for: Knowledge, attitudes, and practices regarding venous thromboembolism prevention in patients with lung cancer
Source: Front Public Health. 2026 Oct 13;14:1980978. doi: 10.3389/fpubh.2026.1980978 (PMC13710242; doi:10.3389/fpubh.2026.1980978)
Supplement: Supplementary file 1 [file Table_1.docx]

**Table S1. Univariable logistic regression analyses for adequate knowledge, positive attitude, and good preventive practice.**

| **Variables** | **Knowledge** | | **Attitude** | | **Practice** | |
| --- | --- | --- | --- | --- | --- | --- |
|  | **OR (95%CI)** | ***P*** | **OR (95%CI)** | ***P*** | **OR (95%CI)** | ***P*** |
| **Knowledge** |  |  | **1.178 (1.081,1.284)** | **<0.001** | **1.240 (1.142,1.348)** | **<0.001** |
| **Attitude** |  |  |  |  | **1.274 (1.157,1.404)** | **<0.001** |
| **Gender** |  |  |  |  |  |  |
| Male | ref |  | ref |  | ref |  |
| Female | 1.191 (0.715,1.982) | 0.502 | 0.932 (0.498,1.744) | 0.825 | 1.597 (0.903,2.825) | 0.108 |
| **Age, years** |  |  |  |  |  |  |
| <40 | ref |  |  |  | ref |  |
| 40~59 | 1.31 (0.607,2.826) | 0.492 | 1.913 (0.762,4.802) | 0.167 | 1.821 (0.772,4.292) | 0.171 |
| 60~74 | 0.657 (0.309,1.398) | 0.275 | 1.201 (0.506,2.852) | 0.678 | 0.736 (0.331,1.635) | 0.451 |
| ≥75 | 0.89 (0.306,2.593) | 0.832 | 1.518 (0.411,5.607) | 0.531 | 1.477 (0.438,4.978) | 0.529 |
| **Residence** |  |  |  |  |  |  |
| Urban | ref |  | ref |  | ref |  |
| Rural or Suburban | **0.489 (0.277,0.861)** | **0.013** | 1.318 (0.642,2.703) | 0.452 | 0.590 (0.326,1.066) | 0.080 |
| **Marital Status** |  |  |  |  |  |  |
| Unmarried | ref |  | ref |  | ref |  |
| Married | 1.473 (0.560,3.873) | 0.433 | 1.538 (0.521,4.544) | 0.436 | 1.167 (0.42,3.24) | 0.767 |
| Others | 1.250 (0.196,7.956) | 0.813 | 1.923 (0.178,20.819) | 0.591 | -- | 0.999 |
| **Education level** |  |  |  |  |  |  |
| Primary or below | ref |  | ref |  | ref |  |
| Junior high school | 0.588 (0253,1.366) | 0.217 | 0.694 (0.232,2.074) | 0.513 | 0.952 (0.408,2.224) | 0.910 |
| Senior high school | 1.140 (0.493,2.636) | 0.759 | 0.955 (0.301,3.022) | 0.937 | 2.5 (0.970,6.443) | 0.058 |
| Associate degree | 2.456 (0.976,6.180) | 0.056 | 0.378 (0.127,1.126) | 0.081 | 1.875 (0.715,4.914) | 0.201 |
| Bachelor's or above | 1.667 (0.742,3.745) | 0.216 | 0.758 (0.259,2.218) | 0.612 | 1.797 (0.76,4.247) | 0.182 |
| **Occupation** |  |  |  |  |  |  |
| Farmer | ref |  | ref |  | ref |  |
| Worker | 1.045 (0.498,2.193) | 0.907 | 0.488 (0.197,1.208) | 0.121 | 2.182 (0.954,4.991) | 0.065 |
| Civil servant | **2.832 (1.272,6.304)** | **0.011** | 0.985 (0.351,2.768) | 0.978 | **2.593 (1.081,6.218)** | **0.033** |
| Self-employed | 1.370 (0.569,3.300) | 0.483 | 1.554 (0.399,6.051) | 0.525 | 2.444 (0.88,6.789) | 0.086 |
| Retired | 1.425 (0.701,2.896) | 0.328 | 0.454 (0.191,1.082) | 0.075 | 1.37 (0.655,2.869) | 0.403 |
| **Medical insurance** |  |  |  |  |  |  |
| Yes | ref |  | ref |  | ref |  |
| No | **0.340 (0.116,0.997)** | **0.049** | 0.593 (0.199,1.770) | 0.349 | 0.442 (0.163,1.195) | 0.108 |
| **Other Comorbidities** |  |  |  |  |  |  |
| 0 | ref |  | ref |  | ref |  |
| 1~2 | 0.906 (0.533,1.538) | 0.714 | 1.431 (0.709,2.889) | 0.318 | 0.932 (0.516,1.684) | 0.817 |
| ≥3 | **0.331 (0.119,0.917)** | **0.033** | **0.253 (0.095,0.673)** | **0.006** | **0.297 (0.113,0.780)** | **0.014** |
| **History of thrombosis** |  |  |  |  |  |  |
| Yes | ref |  | ref |  | ref |  |
| No | 0.833 (0.405,1.716) | 0.621 | 1.178 (0.499,2.780) | 0.708 | 0.948 (0.430,2.092) | 0.896 |
| **Activity status (past 3 days)** |  |  |  |  |  |  |
| Independent daily living | ref |  | ref |  | ref |  |
| Require assistance | 1.631 (0.583,4.564) | 0.351 | 0.822 (0.256,2.64) | 0.742 | 2.047 (0.570,7.357) | 0.272 |
| Completely bedridden | 0.297 (0.030,2.895) | 0.296 | 0.758 (0.077,7.465) | 0.813 | 1.316 (0.134,12.88) | 0.813 |
| **Ever learned VTE** |  |  |  |  |  |  |
| Yes | ref |  | ref |  | ref |  |
| No | **0.320 (0.187,0.548)** | **<0.001** | 0.675 (0.352,1.293) | 0.236 | **0.518 (0.289,0.929)** | **0.027** |
| **Primary caregiver** |  |  |  |  |  |  |
| Children | ref |  | ref |  | ref |  |
| Spouse | 0.825 (0.431,1.579) | 0.561 | 1.123 (0.498,2.533) | 0.779 | 0.586 (0.287,1.197) | 0.142 |
| Parents | **2.273 (1.029,5.022)** | **0.042** | 1.008 (0.394,2.579) | 0.986 | 1 (0.417,2.401) | 1 |
| Rotating care | 2.386 (0.670,8.499) | 0.180 | 1.426 (0.283,7.186) | 0.667 | 1.833 (0.369,9.112) | 0.459 |
| Other | 1.525 (0.688,3.379) | 0.299 | 0.752 (0.297,1.903) | 0.547 | 0.667 (0.281,1.58) | 0.357 |
| **Reason for hospitalization** |  |  |  |  |  |  |
| Lung cancer surgery | ref |  | ref |  | ref |  |
| Systemic cancer therapy | 1.453 (0.749,2.816) | 0.269 | **2.974 (1.286,6.878)** | **0.011** | 1.214 (0.602,2.449) | 0.588 |
| Complication/symptom management | 1.673 (0.592,4.731) | 0.332 | 7.795 (0.961,63.234) | 0.055 | 2.991 (0.777,11.50) | 0.111 |
| Routine follow‑up | 1.181 (0.582,2.395) | 0.645 | 0.957 (0.441,2.077) | 0.912 | 1.319 (0.617,2.822) | 0.475 |
