# Supplementary material for: Knowledge, attitudes, and practices regarding venous thromboembolism prevention in patients with lung cancer
Source: Front Public Health. 2026 Oct 13;14:1980978. doi: 10.3389/fpubh.2026.1980978 (PMC13710242; doi:10.3389/fpubh.2026.1980978)
Supplement: Supplementary file 2 [file Table_2.docx]

**Questionnaire on Knowledge, Attitude, and Practice (KAP) Regarding Venous Thromboembolism Prevention**

**Dear Patient,**

Greetings from our pharmacy team. Venous thromboembolism (VTE, commonly known as “blood clots”) is a frequent complication in patients with cancer, and timely prevention is of great importance. To better understand your awareness, perceptions, and daily practices regarding VTE prevention, we are conducting this survey. Your responses will help us optimize patient education and provide more precise and practical VTE prevention services in the future. We solemnly promise that this questionnaire is anonymous and does not involve any personal privacy. All data will be used only for statistical analysis and will not be disclosed to the public. It will take approximately 8–10 minutes to complete. Please answer based on your actual situation. Your honest feedback is extremely valuable to us. Thank you for taking the time to support our work during your treatment. We wish you a speedy recovery and all the best!

**I. Demographic Information**

1. Your gender:
A. Male
B. Female

2. Your age (years):
A. <40
B. 40–59
C. 60–74
D. ≥75

3. Your current residence:
A. Urban
B. Rural

4. Your marital status:
A. Unmarried
B. Married
C. Other

5. Your highest educational level:
A. Primary school or below
B. Junior high school
C. Senior high school / technical secondary school
D. Associate degree
E. Bachelor’s degree or above

6. Your occupation:
A. Farmer
B. Worker
C. Civil servant or public institution employee
D. Self-employed
E. Retired

7. Do you use medical insurance to pay for medical expenses?
A. Yes
B. No

8. Do you have any other chronic diseases?
A. Yes
B. No

9. Do you have a history of thrombosis?
A. Yes
B. No

10. Your activity status in the past three days:
A. Independent in daily living, able to ambulate freely
B. Require assistance, able to ambulate for some time each day
C. Completely bedridden, unable to ambulate

11. Have you ever learned about venous thromboembolism (VTE) through any channel?
A. Yes
B. No

12. Your primary caregiver during your most recent hospitalization:
A. Children
B. Spouse
C. Parents
D. Rotating care
E. Other

13. The main reason for your current hospitalization or the treatment you are receiving:
A. Lung cancer‑related surgery
B. Systemic anti‑cancer therapy (e.g., chemotherapy, targeted therapy)
C. Management of complications or symptoms
D. Routine follow‑up

**II. Knowledge**

1. Venous thromboembolism (VTE) mainly includes deep vein thrombosis and pulmonary embolism.
A. True B. False C. Uncertain

2. Hypercoagulable state (blood that clots more easily than usual) is one of the main causes of VTE.
A. True B. False C. Uncertain

3. VTE can present as unilateral lower extremity swelling and pain.
A. True B. False C. Uncertain

4. Pulmonary embolism can present as dyspnea, chest pain, and hemoptysis, and can be life‑threatening when severe.
A. True B. False C. Uncertain

5. Cancer increases the risk of VTE.
A. True B. False C. Uncertain

6. Prolonged sitting or bed rest increases the risk of VTE.
A. True B. False C. Uncertain

7. Dislodgement of a deep vein thrombus may cause pulmonary embolism, which can be fatal.
A. True B. False C. Uncertain

8. Do you think VTE can be prevented?
A. Yes B. No C. Uncertain

9. Wearing compression stockings, using pneumatic compression devices, or using anticoagulants can prevent VTE.
A. True B. False C. Uncertain

10. VTE prevention/treatment (especially with anticoagulants) may increase the risk of bleeding, requiring monitoring and timely reporting of abnormal bleeding.
A. True B. False C. Uncertain

11. Which of the following conditions should prompt you to notify healthcare providers to rule out pulmonary embolism?
A. Sudden dyspnea/chest pain
B. Mild low back pain
C. Occasional hiccups
D. Loss of appetite

12. When is the recommended time to wear medical compression stockings?
A. Before getting out of bed in the morning
B. Before going to sleep
C. Any time
D. The tighter, the better

13. Which of the following bleeding signs is more indicative of “need for prompt reporting”?
A. Minor skin scratch
B. Melena/hematemesis
C. Mild dry mouth
D. Mild headache

**III. Attitude**

1. I worry that I might develop VTE.
A. Strongly disagree B. Disagree C. Neutral D. Agree E. Strongly agree

2. I worry that VTE will affect my physical health.
A. Strongly disagree B. Disagree C. Neutral D. Agree E. Strongly agree

3. I worry that developing VTE will impose a financial burden on my family.
A. Strongly disagree B. Disagree C. Neutral D. Agree E. Strongly agree

4. I worry that using anticoagulants may cause bleeding, so I might be reluctant to use them.
A. Strongly disagree B. Disagree C. Neutral D. Agree E. Strongly agree

5. I find compression stockings or pneumatic compression devices uncomfortable/troublesome, so I find it difficult to adhere to their use.
A. Strongly disagree B. Disagree C. Neutral D. Agree E. Strongly agree

6. I believe that VTE prevention will bring health benefits to me.
A. Strongly disagree B. Disagree C. Neutral D. Agree E. Strongly agree

7. If I develop VTE, I will actively cooperate with healthcare providers for treatment and follow‑up.
A. Strongly disagree B. Disagree C. Neutral D. Agree E. Strongly agree

8. I am confident that I can comply with the measures for VTE prevention.
A. Strongly disagree B. Disagree C. Neutral D. Agree E. Strongly agree

**IV. Practice**

(Please select the option that best reflects your actual situation over the past month.)

1. Do you actively ask doctors, nurses, or pharmacists about knowledge of VTE prevention?
A. Never B. Rarely C. Sometimes D. Often E. Always

2. When permitted by the doctor, do you get out of bed and ambulate or increase walking as early as possible?
A. Never B. Rarely C. Sometimes D. Often E. Always

3. Do you comply with the use of medical graduated compression stockings for VTE prevention as prescribed?
A. Never B. Rarely C. Sometimes D. Often E. Always

4. Can you adhere to oral or injectable anticoagulant therapy for VTE prevention as prescribed?
A. Never B. Rarely C. Sometimes D. Often E. Always

5. During anticoagulant therapy, do you proactively monitor adverse reactions (e.g., gingival bleeding, conjunctival congestion, melena, etc.) and promptly inform the doctor, nurse, or pharmacist if any occur?
A. Never B. Rarely C. Sometimes D. Often E. Always
